# Supplementary material for: Development and Application of a Senolytic Predictor for Discovery of Novel Senolytic Compounds and Herbs
Source: Molecules. 2025 Jun 19;30(12):2653. doi: 10.3390/molecules30122653 (PMC12196162; doi:10.3390/molecules30122653)
Supplement: Supplementary file 1 [file molecules-30-02653-s001.zip › Supplementary_figures.pdf]

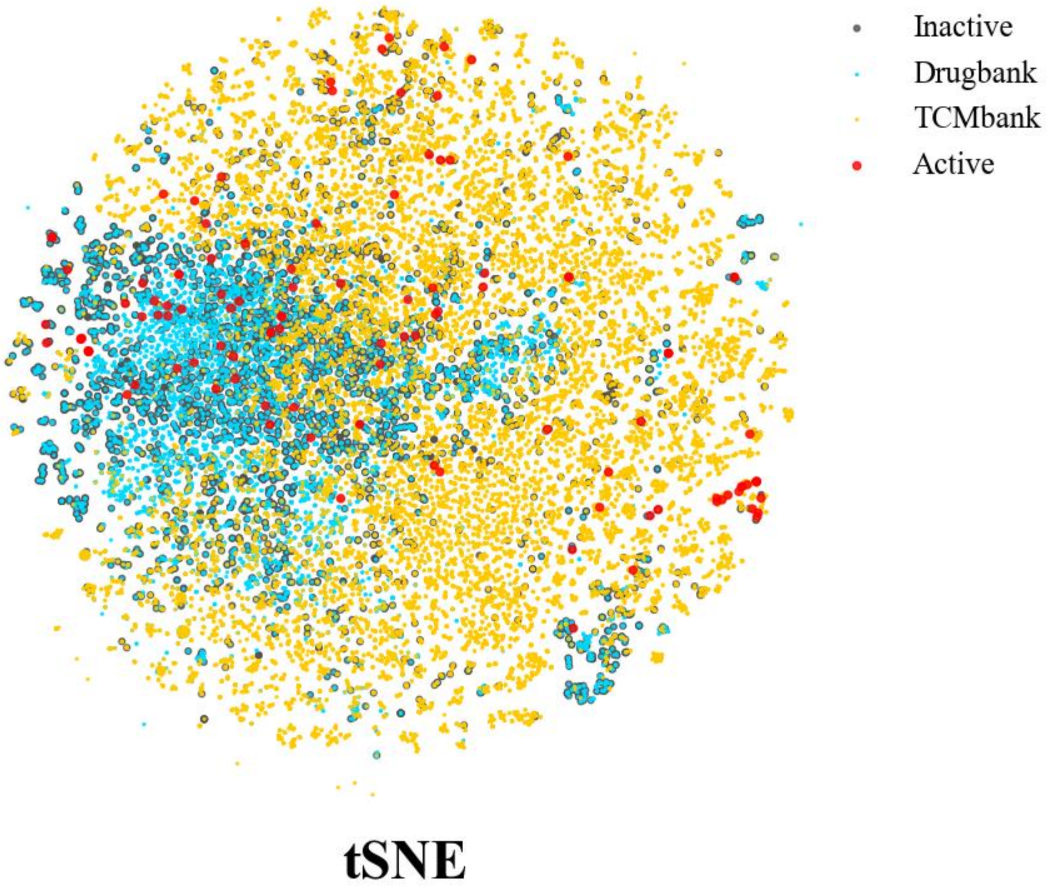

Supplementary Fig S1 t-SNE of the chemical spatial distribution of all compounds in the training dataset, Drugbank and TCMbank datasets. Each point represents a compound, gray points indicate negative compounds in the training set, blue points indicate compounds in Drugbank, yellow points indicate compounds in TCMbank, and red points indicate active compounds in the training set.

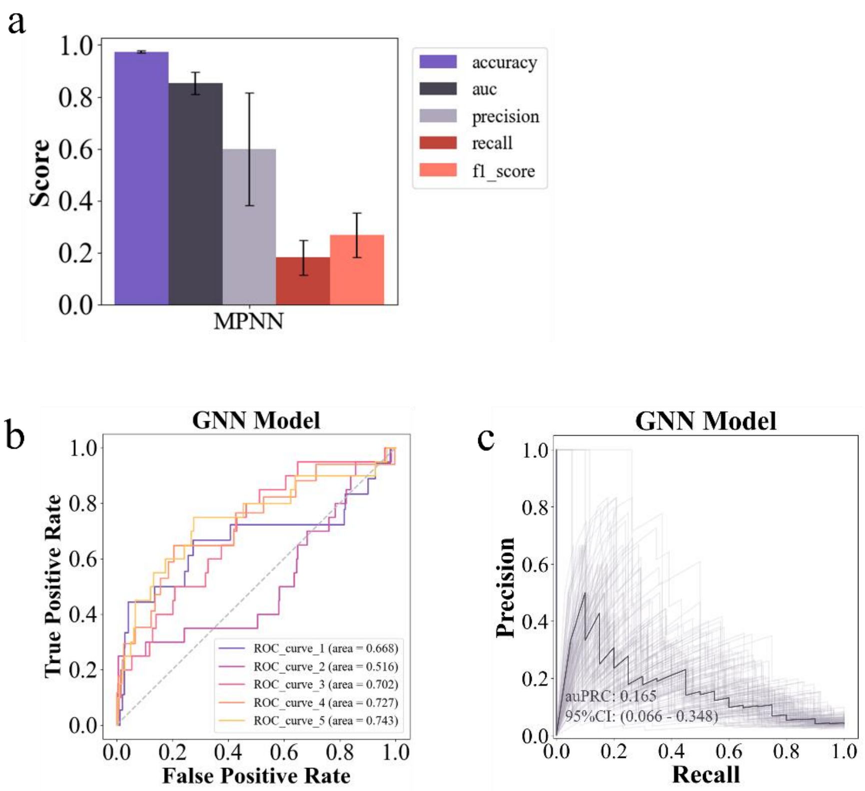

Supplementary Fig S2 Performance plot of the MPNN and GNN model. **a** Performance plot of the message passing graph neural network (MPNN) model. The values of the evaluation metrics are the average of 20 iterations of repeated random split cross-validation, including accuracy, AUC, precision, recall, and f1_score. **b, c** AUC curves and precision-recall curves for the graph neural network (GNN) model.

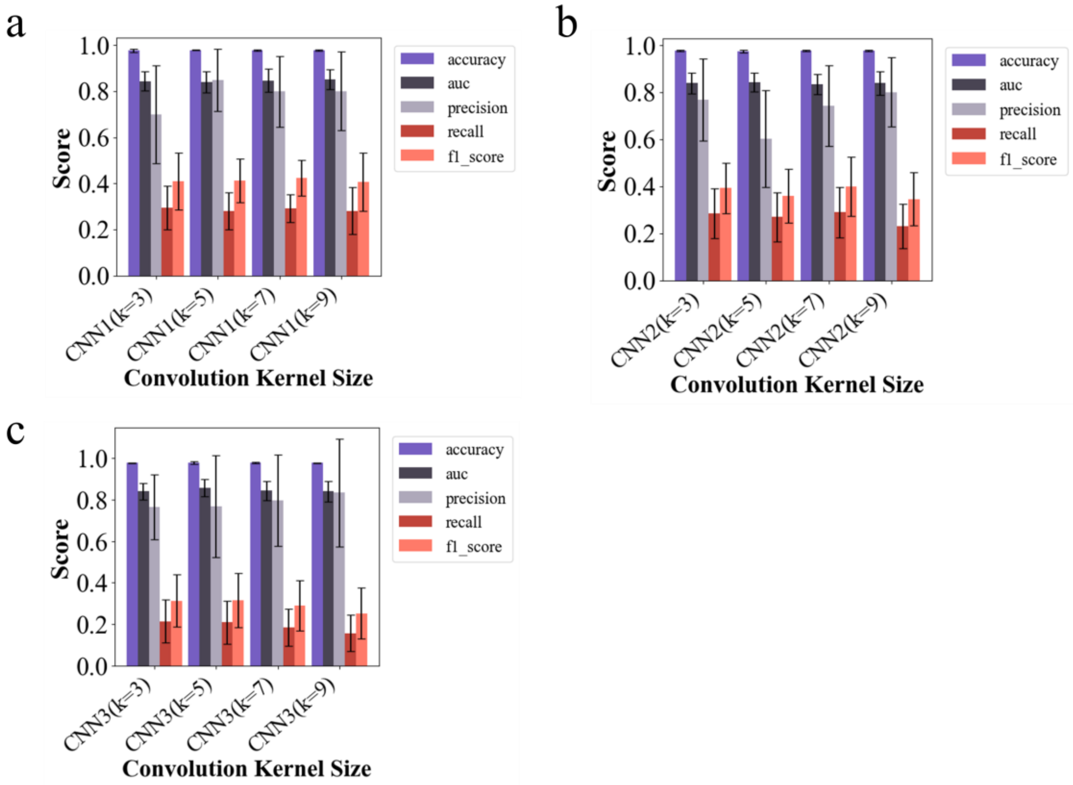

Supplementary Fig S3 Performance plot of the convolutional neural network model with defferent convolution kernel size . (**a-c**) The values of the evaluation metrics are the average of 20 iterations of repeated random split cross-validation, including accuracy, AUC, precision, recall, and f1_score. CNN1, CNN2, and CNN3 indicate that the model has 1, 2, and 3 convolutional layers, respectively.The k=3, 5, 7, 9 indicate that the kernel sizes for the first convolutional layer in CNN models.

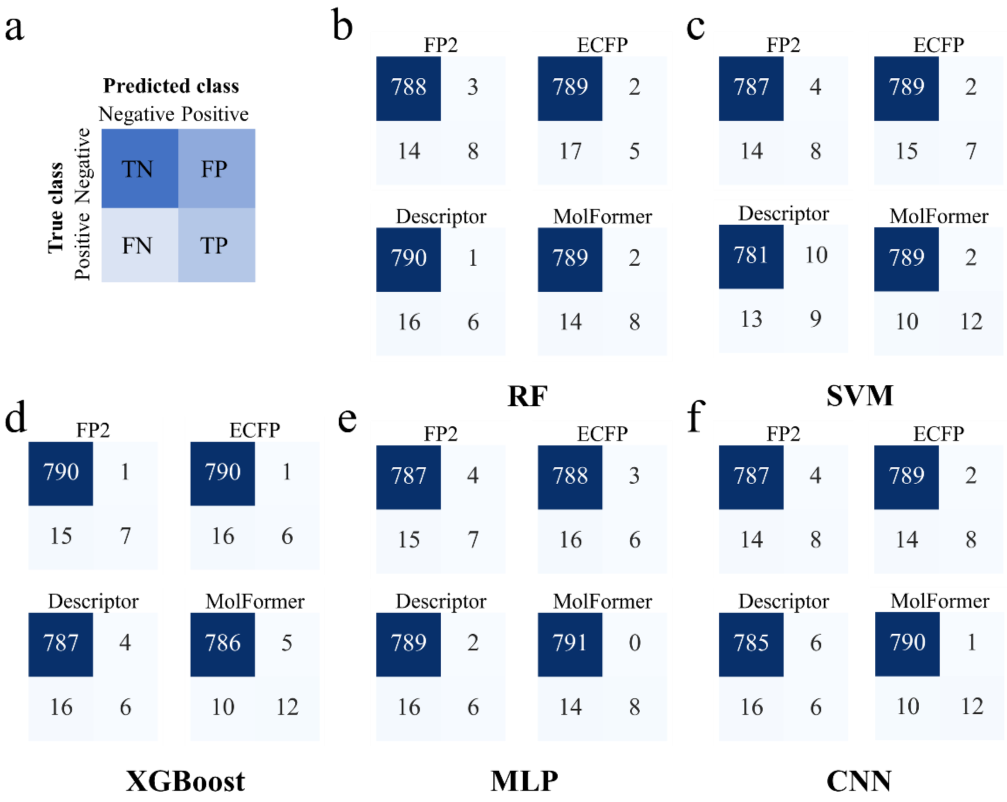

Supplementary Fig S4 Confusion matrix. **a.** Schematic diagram of the confusion matrix. **b-f** correspond to the confusion matrix plots for each model in Fig. 2. The best performing model in the training data set with 20 random splits is selected to plot the confusion matrix. Divide 20% of the data in the training set into a test set to show the prediction performance on the model test set.

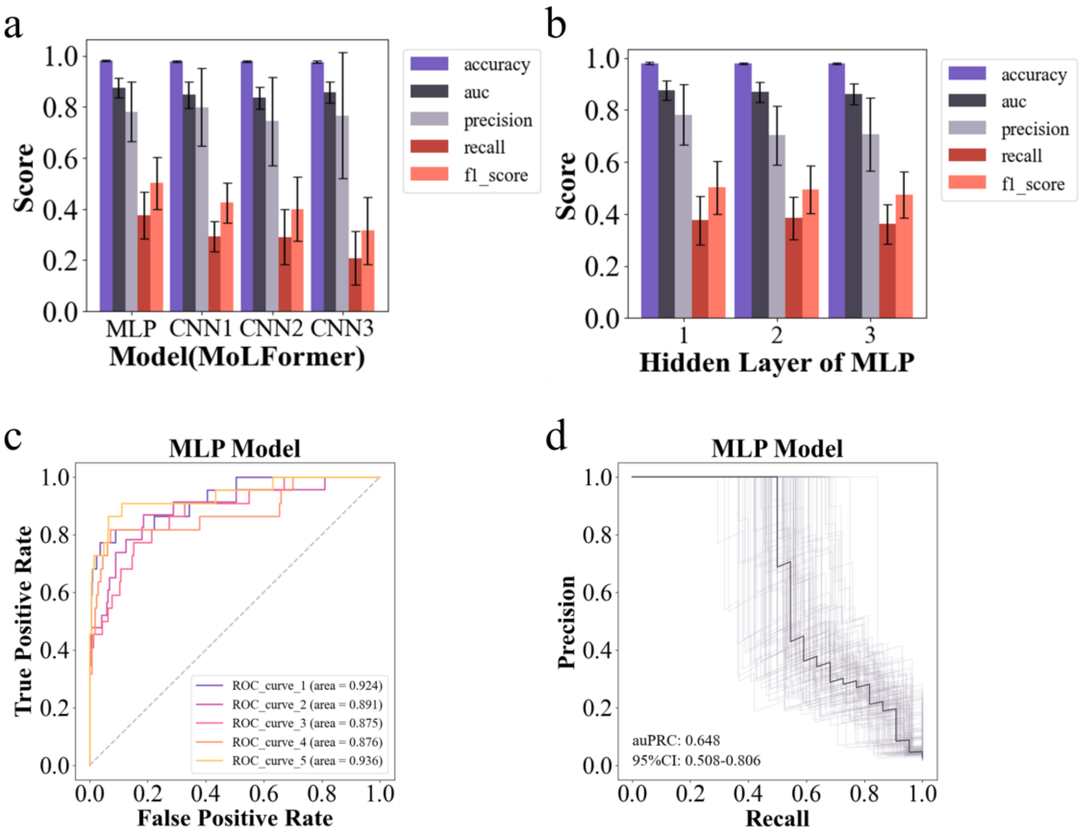

Supplementary Fig S5 The CNN and MLP model tuning. **a** Performance plot of the convolutional neural network model. The values of the evaluation metrics are the average of 20 iterations of repeated random split cross-validation, including accuracy, AUC, precision, recall, and f1_score. CNN1, CNN2, and CNN3 indicate that the model has 1, 2, and 3 convolutional layers, respectively, while the MLP has no convolutional layers. **b** Performance plot of the MLP model. The evaluation metrics are the same as in **a**. 1, 2 and 3 denote the number of hidden layers of the MLP model. **c, d** AUC curves and precision-recall curves for 10 MLP models generated using ensemble.

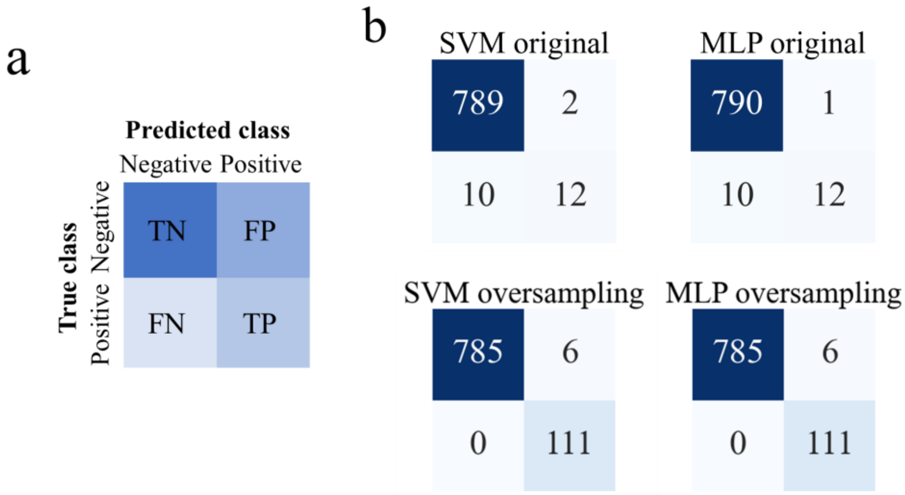

Supplementary Fig S6 Confusion matrix. **a** Schematic diagram of the confusion matrix. **b** correspond to the confusion matrix plots for each model in Fig. 3g. The best performing model in the training data set with 20 random splits is selected to plot the confusion matrix. Divide 20% of the data in the training set into a test set to show the prediction performance on the model test set.

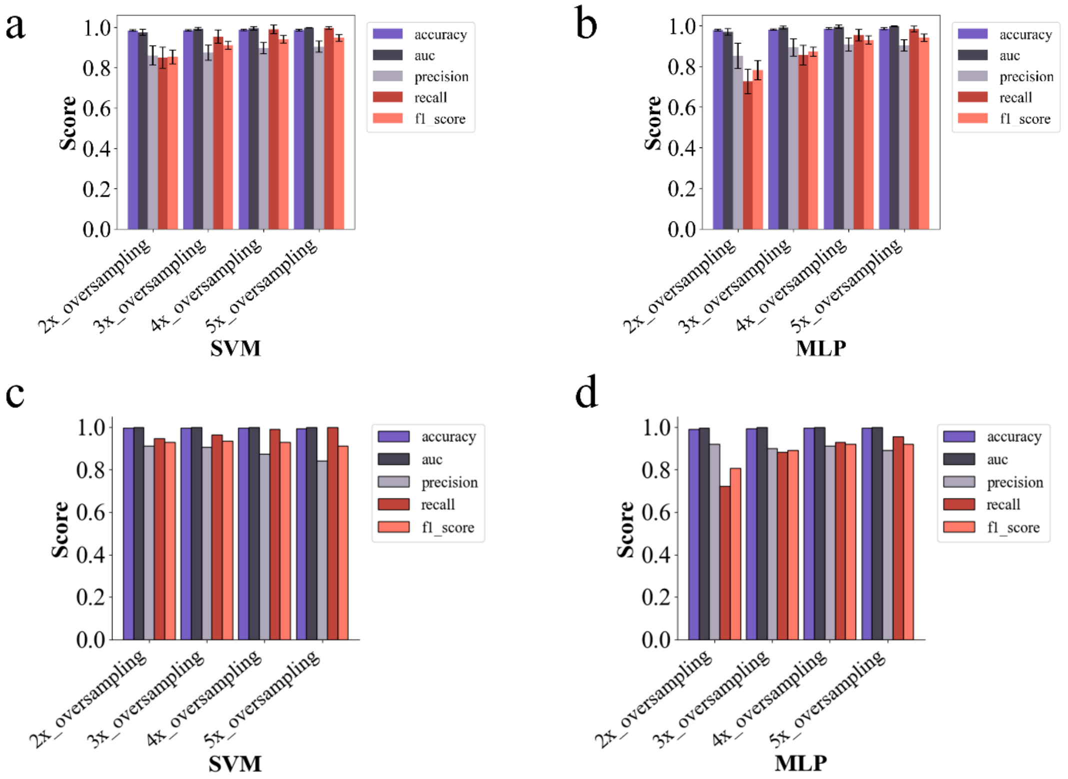

Supplementary Fig S7 MolFormer model-based oversampling. **a, b** Performance plot of the SVM and MLP model on the test set. Positive samples were oversampled using the MolFormer model, with 2-, 3-, 4-, and 5-fold oversampling employed. The values of the evaluation metrics are the average of 20 iterations of repeated random split cross-validation, including accuracy, AUC, precision, recall, and f1_score. **c, d** Performance plot of the SVM and MLP model on the full training set. The training set features used for testing are generated by the MolFormer model under a new random seed, different from the feature representation used for training. Consistent with plots a,b, the horizontal coordinates represent 2-, 3-, 4-, and 5-fold oversampling, respectively

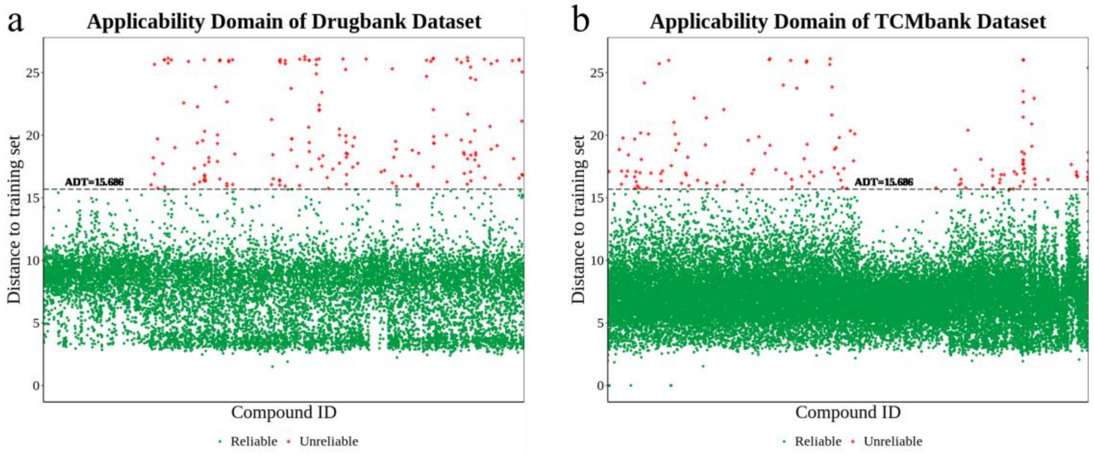

Supplementary Fig S8 Analysis of the Applicability domain. **a, b** Applicability domain scatter plots of compounds in the Drugbank and TCMbank datasets, respectively. The applicability domain threshold (ADT) is 15.686, and each point represents a compound, with green points indicating that the compound is within the AD and red points indicating that the compound is outside the AD.
